# Down Syndrome: Changing Cardiac Phenotype?

**DOI:** 10.1542/peds.2016-1223

**Published:** 2016-06-01

**Authors:** Tiffany Riehle-Colarusso, Matthew E. Oster

**Affiliations:** a Centers for Disease Control and Prevention, National Center on Birth Defects and Developmental Disabilities, Atlanta, Georgia; b Children's Healthcare of Atlanta, Emory University School of Medicine, Atlanta, Georgia

Down syndrome (DS) is the most common chromosomal abnormality, affecting 1 in 700 infants born yearly in the United States.^1^ The birth prevalence of DS varies internationally among populations, likely due to variations in maternal age, race/ethnicity, use of prenatal screening, and terminations of affected pregnancies.^2, 3^ Approximately half of all infants born with DS also have a congenital heart defect (CHD), the most common type being atrioventricular septal defect (AVSD), with rates from 30% to 60%.^4^ In the era of increasing maternal age and pregnancy terminations, ^2^ it is interesting to investigate if and how the pattern of associated CHDs among infants with DS has changed.

In this issue of *Pediatrics*, Bergstrom et al^5^ describe the pattern of CHDs among Swedish liveborn infants with DS from 1992 to 2012. In this nationwide, population-based cohort, over half had a CHD; the 3 most common being AVSD, ventricular septal defect, and atrial septal defect. Although the overall rate and risk for CHDs remained constant, differences were noted among CHD phenotypes over time. When adjusting for several factors, the risk of complex CHDs decreased by 40% from birth period 1992–1994 to birth period 2010–2012, with a concurrent rise in less severe CHDs. Among livebirths with DS and CHDs, the rate of AVSD decreased from 46% to 30%, whereas the rate of ventricular septal defect doubled from 14% to 31%. The authors hypothesize this phenotypic shift could be the result of improved prenatal detection, especially among older mothers, leading to increased pregnancy termination.^5^

The finding of a phenotypic shift to less complex CHDs is intriguing but should be interpreted cautiously. First, although this is a large population-based study of DS livebirths, it lacked information on prenatal screening or pregnancy terminations. Understanding the true occurrence of CHDs would include not only livebirths, but also stillbirths and terminations of pregnancy. Thus, it is unclear whether the decreased rate of AVSD among livebirths was due to increased pregnancy terminations or a change in the true occurrence of CHDs in all fetuses with DS. However, a French study by Stoll et al^4^ of all outcomes from DS-affected pregnancies over 26 years revealed a similar distribution of AVSD (30%) among those with CHDs, but did not investigate changes over time.^4^

Second, differences in underlying prevalence of both DS and CHDs across populations might limit the generalizability of the current findings. Documented prevalence variability is likely due to differences in study design, population characteristics (eg, maternal age or race/ethnicity), time period, availability of prenatal care, and pregnancy terminations. For example, in the United States, racial/ethnic differences in prevalence and survival of children with DS, CHDs, or both have been observed, likely due to a multifactorial interaction as yet to be elucidated.^3, 6–8^ In Cocchi et al, ^2^ the stable birth prevalence of DS despite an increase in older mothers was attributed to the increased use of prenatal diagnoses and pregnancy terminations. Similarly, in this *Pediatrics* study, Bergstrom et al^5^ hypothesized that a 1% decreased risk of a CHD for every year of maternal age might be associated with more careful prenatal assessment and pregnancy termination. Although the percentage of DS births to older mothers was reported to be increasing internationally, the increase was much smaller in North American countries compared with Europe.^2^ A lower and more stable rate of pregnancy terminations in North America was also reported.^2^ Access to prenatal health care in the United States differs from Sweden, thereby potentially affecting the prenatal detection of CHDs and pregnancy terminations in the United States. Together these country-specific differences could mean that a shift in cardiac phenotype among DS livebirths may not be as evident in the United States as in Sweden.

Although the survival of children born with DS and CHDs has greatly improved, these children are still at higher risk for mortality, ^7^ health care cost, ^6^ and neurodevelopmental delay^9^ compared with children born without birth defects. For example, in guidelines to optimize neurodevelopmental outcome for children with CHDs, children with DS and CHDs are at high-risk for developmental delay.^9^ If there is a decreasing trend in complex CHDs, then perhaps children with DS and CHDs might have better outcomes than previous generations. Understanding the phenotypic expression of DS is helpful for resource planning and targeting health care needs. Health supervision guidelines used by pediatricians who care for children with DS note that early intervention may improve outcomes and functioning.^10^

CHDs are 1 of the most serious conditions associated with DS, but there are other comorbidities. More research is needed to evaluate whether other common conditions such as hearing loss, obstructive sleep apnea, eye disease, cancer, or autoimmune diseases have changed in persons with DS, which could signal an improvement in overall health outcomes for this population.

## Abbreviations

AVSD: atrioventricular septal defect
CHD: congenital heart defect
DS: Down syndrome
